# Epidemiological and Clinical Profile of Heart Failure at the Morafeno University Hospital of Toamasina, Madagascar: A Three-Year Retrospective Study

**DOI:** 10.7759/cureus.109536

**Published:** 2026-05-24

**Authors:** Rado Olivier Rakoto Sedson, Jean Christian Bezara, Holy Mihanta Sabrina Ranaivoson, Mamisoa Herinirina Nomenjanahary, Narindrarimanana Avisoa Randriamihangy, Nirina Rabearivony, Solofonirina Rakotoarimanana

**Affiliations:** 1 Faculty of Medicine, University of Toamasina, Toamasina, MDG; 2 Faculty of Medicine, University of Fianarantsoa, Fianarantsoa, MDG; 3 Faculty of Medicine, University of Mahajanga, Mahajanga, MDG; 4 Department of Cardiology, Mahavoky Atsimo University Hospital, Mahajanga, MDG; 5 Faculty of Medicine, University of Antananarivo, Antananarivo, MDG

**Keywords:** cardiovascular risk factors, clinical profile, echocardiography, heart failure, hospitalization, madagascar, prognosis, sub-saharan africa

## Abstract

Background

Heart failure (HF) represents a major public health challenge worldwide, with particularly high morbidity and mortality in resource-limited settings. Despite its growing burden in sub-Saharan Africa, comprehensive epidemiological data from Madagascar remain scarce. This study aimed to describe the epidemiological, clinical, paraclinical, therapeutic, and prognostic profile of patients hospitalized for heart failure at the Centre Hospitalier Universitaire Morafeno Toamasina (University Hospital Center Morafeno Toamasina) (CHUMT), and to identify independent factors associated with prolonged hospitalization, rehospitalization, and in-hospital mortality.

Methods

We conducted a retrospective, cross-sectional, descriptive study in the cardiology department of CHUMT from January 1, 2021, to December 31, 2023. All patients hospitalized for echocardiographically confirmed heart failure were included. Data were collected from medical records using standardized forms. Binary logistic regression analyses (univariate followed by multivariate) were performed using Stata version 13 (StataCorp, College Station, USA) to identify independent factors associated with prolonged hospitalization (≥7 days), rehospitalization during the study period, and in-hospital mortality. Statistical significance was set at p<0.05.

Results

A total of 168 patients were included over the 36-month study period. The mean age was 54 ± 14 years (range: 18-89 years), with a sex ratio of 0.97 (50.6% female). The most prevalent cardiovascular risk factors were hypertension (n=86; 51.2%), overweight (n=46; 27.4%), and diabetes mellitus (n=37; 22.0%). Global heart failure was the predominant clinical form (n=127; 75.6%). Ischemic cardiomyopathy was the leading etiology (n=46; 27.4%), followed by hypertensive heart disease (n=39; 23.2%). Heart failure with reduced ejection fraction (HFrEF, left ventricular ejection fraction (LVEF) <40%) accounted for 53.6% (n=90) of cases. Clinical outcomes included favorable discharge in 81.5% (n=137) of cases, in-hospital mortality of 9.5% (n=16), and a 6-month rehospitalization rate of 41.7% (n=70). In multivariate analysis, right ventricular systolic dysfunction was the only independent factor associated with prolonged hospitalization (adjusted OR = 6.1; 95% CI: 1.5-23.5; p = 0.009). Atrial fibrillation (adjusted OR = 3.4; 95% CI: 1.3-29.3; p = 0.036) and intraventricular conduction disorders (adjusted OR = 2.3; 95% CI: 1.4-36.6; p = 0.013) were independently associated with rehospitalization. No factor reached statistical significance for in-hospital mortality in multivariate analysis.

Conclusion

Heart failure at CHUMT affects relatively young patients, predominantly presenting with global heart failure and primarily attributable to ischemic and hypertensive etiologies. Right ventricular systolic dysfunction, atrial fibrillation, and intraventricular conduction disorders are key independent prognostic factors. Strengthening cardiovascular prevention strategies, improving early diagnosis, and implementing structured therapeutic patient education programs are essential to reduce mortality, hospitalization duration, and readmission rates in this resource-limited setting.

## Introduction

Heart failure (HF) represents one of the most significant cardiovascular public health challenges of the 21st century, affecting an estimated 64 million individuals worldwide [1,2]. This syndrome, characterized by the heart’s inability to pump sufficient blood to meet the body’s metabolic demands, is associated with substantial morbidity, mortality, and healthcare costs across all geographic regions [3]. The global burden of heart failure continues to rise, driven by aging populations, improved survival from acute coronary syndromes, and the increasing prevalence of cardiovascular risk factors such as hypertension, diabetes mellitus, and obesity [1,2].

The epidemiological landscape of heart failure differs markedly between high-income countries and resource-limited settings, particularly in sub-Saharan Africa. While European and North American populations typically present with heart failure at advanced ages (mean age >70 years) with ischemic heart disease as the predominant etiology [3,4], African patients tend to be significantly younger (mean age 50-60 years) and present with hypertensive heart disease, cardiomyopathies, and rheumatic heart disease as leading causes [5,6]. Furthermore, in-hospital mortality rates in sub-Saharan Africa remain substantially higher than in developed countries, ranging from 9% to 20% compared to 4-7% in Europe [3,5,7].

In Madagascar, cardiovascular diseases constitute an emerging public health priority, yet comprehensive epidemiological data on heart failure remain limited. Previous studies from the capital city of Antananarivo have identified treatment discontinuation and infections as major decompensation factors, but detailed characterization of heart failure phenotypes, prognostic factors, and outcomes in other regions of Madagascar is lacking. The Centre Hospitalier Universitaire Morafeno Toamasina (University Hospital Center Morafeno Toamasina) (CHUMT), located in Madagascar’s second-largest city and principal port, serves as the main tertiary referral center for the eastern region of the country, providing specialized cardiology services to a catchment population of approximately 1.5 million inhabitants.

Understanding the local epidemiological and clinical profile of heart failure is essential for developing targeted prevention strategies, optimizing therapeutic approaches, and allocating healthcare resources effectively in resource-constrained settings. Furthermore, identifying independent prognostic factors associated with adverse outcomes can guide risk stratification and inform clinical decision-making.

Study objectives

The primary objectives of this study were (1) to describe the epidemiological, clinical, paraclinical, therapeutic, and prognostic profile of patients hospitalized for heart failure at CHUMT over a three-year period (2021-2023), and (2) to identify independent factors associated with prolonged hospitalization (≥7 days), rehospitalization during the study period, and in-hospital mortality through multivariate analysis.

This study represents the first comprehensive characterization of heart failure at CHUMT and aims to provide evidence-based insights to inform cardiovascular health policy and clinical practice in Madagascar.

## Materials and methods

Study design and setting

We conducted a retrospective, cross-sectional, descriptive study in the cardiology department of CHUMT, Madagascar. CHUMT is a tertiary care teaching hospital and the primary referral center for cardiovascular diseases in the Atsinanana region of eastern Madagascar. The cardiology department comprises a 20-bed inpatient unit equipped with electrocardiography, transthoracic echocardiography, and basic laboratory facilities.

The study period extended from January 1, 2021, to December 31, 2023, encompassing 36 consecutive months of patient admissions.

Study population

All patients hospitalized in the cardiology department during the study period with a diagnosis of heart failure confirmed by transthoracic echocardiography were eligible for inclusion. Heart failure was defined according to the 2021 European Society of Cardiology (ESC) guidelines as a clinical syndrome characterized by typical symptoms (dyspnea, orthopnea, fatigue) and signs (elevated jugular venous pressure, peripheral edema, pulmonary rales) resulting from structural and/or functional cardiac abnormalities documented by echocardiography [1].

Patients were excluded if they had incomplete medical records lacking essential clinical or echocardiographic data, transfer to another healthcare facility before clinical stabilization, or absence of echocardiographic confirmation of heart failure.

The inclusion and exclusion criteria applied in this study are summarized in Table 1.

**Table 1:** Inclusion and Exclusion Criteria CHUMT: Centre Hospitalier Universitaire Morafeno Toamasina (University Hospital Center Morafeno Toamasina); ESC: European Society of Cardiology; JVP: jugular venous pressure

Criterion Type	Criterion
Inclusion	Hospitalized in the cardiology department of CHUMT during the study period (January 1, 2021 – December 31, 2023)
Inclusion	Diagnosis of heart failure confirmed by transthoracic echocardiography
Inclusion	Heart failure defined per the 2021 ESC guidelines: typical symptoms (dyspnea, orthopnea, fatigue) and signs (elevated JVP, peripheral edema, pulmonary rales) with structural/functional cardiac abnormalities on echocardiography
Exclusion	Incomplete medical records lacking essential clinical or echocardiographic data
Exclusion	Transfer to another healthcare facility before clinical stabilization
Exclusion	Absence of echocardiographic confirmation of heart failure

Data collection

Data were retrospectively extracted from hospital medical records, including admission notes, daily progress notes, laboratory reports, electrocardiograms, echocardiography reports, and discharge summaries. A standardized data collection form was developed and pilot-tested prior to implementation. Two trained investigators (including the principal investigator) independently reviewed medical records and entered data into a secure electronic database. Discrepancies were resolved through consensus discussion with the senior cardiologist.

Definitions and variables

Sociodemographic Variables

The sociodemographic variables included age, sex, place of residence (urban vs. rural), and occupation.

Cardiovascular Risk Factors

The cardiovascular risk factors included hypertension, defined as systolic blood pressure ≥140 mmHg and/or diastolic blood pressure ≥90 mmHg, or current use of antihypertensive medications; diabetes mellitus, defined as fasting plasma glucose ≥7.0 mmol/L (126 mg/dL), or current use of antidiabetic medications; dyslipidemia, defined as total cholesterol ≥5.2 mmol/L (200 mg/dL) or current use of lipid-lowering therapy; smoking, defined as current or former tobacco use; alcohol consumption, defined as regular alcohol intake (≥3 drinks per week); body mass index (BMI), defined as overweight (25.0-29.9 kg/m²) or obesity (≥30 kg/m²); and sedentary lifestyle, defined as <150 minutes of moderate physical activity per week.

Clinical Variables

The symptoms included dyspnea according to the New York Heart Association (NYHA) functional class (I-IV), as well as orthopnea, paroxysmal nocturnal dyspnea, lower limb edema, fatigue, palpitations, chest pain, and syncope. The clinical forms included left heart failure, right heart failure, or global (biventricular) heart failure. The vital signs measured were heart rate, blood pressure, respiratory rate, and oxygen saturation.

Paraclinical Variables

Electrocardiography (ECG): Findings included cardiac rhythm (sinus rhythm, atrial fibrillation, or other arrhythmias), left ventricular hypertrophy (assessed using the Sokolow-Lyon or Cornell criteria), conduction abnormalities (such as left bundle branch block, right bundle branch block, or intraventricular conduction delay), ST-segment and T-wave changes, and pathological Q waves.

Echocardiography: Findings included left ventricular ejection fraction (LVEF) measured by Simpson’s biplane method; heart failure classification, i.e., HFrEF (LVEF <40%), heart failure with mildly reduced ejection fraction (HFmrEF) (LVEF 40-49%), and heart failure with preserved ejection fraction (HFpEF) (LVEF ≥50%) [1]; left ventricular end-diastolic diameter (LVEDD); right ventricular systolic function (assessed by tricuspid annular plane systolic excursion (TAPSE) and S’ wave velocity); valvular abnormalities (such as mitral regurgitation, tricuspid regurgitation, and aortic stenosis or regurgitation); and pulmonary arterial hypertension (defined as an estimated systolic pulmonary artery pressure greater than 35 mmHg).

Laboratory Tests: Tests included complete blood count (hemoglobin, white blood cell count), serum creatinine and estimated glomerular filtration rate (eGFR), serum electrolytes (sodium, potassium), fasting blood glucose, and C-reactive protein (CRP).

Etiological Classification

Ischemic cardiomyopathy was defined by a history of myocardial infarction and regional wall motion abnormalities on echocardiography. Hypertensive heart disease was identified by long-standing hypertension with left ventricular hypertrophy and/or diastolic dysfunction. Dilated cardiomyopathy was characterized by left ventricular dilation (LVEDD >55 mm) with reduced ejection fraction in the absence of ischemic or valvular disease. Valvular heart disease was defined by significant valvular stenosis or regurgitation. Peripartum cardiomyopathy was diagnosed when heart failure developed in the last month of pregnancy or within 5 months postpartum. Rhythm disorder cardiomyopathy was defined as heart failure secondary to persistent tachyarrhythmia. Cases that did not meet any of these criteria were classified as other or unknown etiologies.

Decompensation Factors

Decompensation was attributed to one or more of the following factors: treatment discontinuation, infections (including pneumonia, urinary tract infection, and sepsis), uncontrolled hypertension, cardiac arrhythmias, dietary non-compliance (excessive salt or fluid intake), anemia, pulmonary embolism, or acute coronary syndrome.

Therapeutic Management

Pharmacological therapy included loop diuretics, angiotensin-converting enzyme inhibitors (ACEIs) or angiotensin receptor blockers (ARBs), beta-blockers, mineralocorticoid receptor antagonists (MRAs), digoxin, anticoagulants, and sodium-glucose cotransporter-2 (SGLT2) inhibitors. Non-pharmacological interventions consisted of oxygen therapy, dietary sodium restriction, and fluid restriction.

Outcome Variables

The following outcome variables were assessed: duration of hospitalization (in days), prolonged hospitalization (defined as a hospital stay of 7 days or more), in-hospital mortality, and rehospitalization, defined as recurrent admissions for heart failure decompensation during the study period.

Statistical analysis

Data were analyzed using Stata statistical software version 13 (StataCorp, College Station, USA). Continuous variables were expressed as mean ± standard deviation (SD) or median with interquartile range (IQR), depending on distribution normality (assessed by the Shapiro-Wilk test). Categorical variables were presented as frequencies and percentages.

To identify factors associated with prolonged hospitalization (≥7 days), rehospitalization, and in-hospital mortality, we performed binary logistic regression analyses. Variables with p<0.20 in univariate analysis were entered into multivariate models using a stepwise backward elimination approach. Results were reported as odds ratios (OR) with 95% confidence intervals (CI). Statistical significance was set at p<0.05 (two-tailed).

Ethical considerations

The study protocol was approved by the institutional ethics committee of the University of Toamasina Faculty of Medicine. As this was a retrospective study using anonymized data from medical records, the requirement for individual informed consent was waived. All data were handled in accordance with principles of medical confidentiality and data protection. Patient identifiers were removed from the database, and each patient was assigned a unique study identification number.

## Results

Epidemiological characteristics

During the 36-month study period from January 1, 2021, to December 31, 2023, a total of 168 patients hospitalized for echocardiographically confirmed heart failure met the inclusion criteria and were enrolled in the study.

Age and Sex Distribution

The mean age of the study population was 54 ± 14 years, with a range from 18 to 89 years. The age distribution revealed that the 40-59 years age group was the most represented, accounting for 42.3% (n=71) of patients, followed by the ≥60 years group (38.1%, n=64) and the 18-39 years group (19.6%, n=33).

The sex distribution showed near parity, with 85 female patients (50.6%) and 83 male patients (49.4%), yielding a sex ratio (male:female) of 0.97.

Geographic and Socioeconomic Characteristics

The majority of patients (71.4%, n=120) resided in urban areas, while 28.6% (n=48) came from rural settings. Regarding occupation, the informal sector represented the largest group (38.1%, n=64), followed by civil servants (21.4%, n=36), retired individuals (15.5%, n=26), formal sector employees (13.1%, n=22), farmers (8.3%, n=14), and unemployed persons (3.6%, n=6).

Cardiovascular Risk Factors

The prevalence of cardiovascular risk factors in the study population is presented in Table 2. Hypertension was the most prevalent risk factor, affecting 51.2% (n=86) of patients, followed by overweight and diabetes mellitus. 

**Table 2:** Cardiovascular Risk Factors in the Study Population (N=168) BMI: body mass index

Risk Factor	n	%
Hypertension	86	51.2
Overweight (BMI 25.0–29.9 kg/m²)	46	27.4
Diabetes mellitus	37	22.0
Active smoking	25	14.9
Dyslipidemia	20	11.9
Sedentary lifestyle	16	9.5
Alcohol consumption	14	8.3
Obesity (BMI ≥30 kg/m²)	12	7.1

Clinical presentation

Symptoms

Dyspnea was the most common presenting symptom, reported in 94.6% (n=159) of patients. Among these, 67.9% (n=114) presented with severe dyspnea classified as NYHA functional class III or IV. Other frequent symptoms included lower limb edema (78.6%, n=132), orthopnea (72.0%, n=121), fatigue (68.5%, n=115), palpitations (34.5%, n=58), chest pain (28.6%, n=48), and syncope (8.3%, n=14).

Clinical Forms

Global (biventricular) heart failure was the predominant clinical presentation, observed in 75.6% (n=127) of patients. Left heart failure was present in 15.5% (n=26) of cases, while isolated right heart failure accounted for 8.9% (n=15) of presentations.

Paraclinical findings

Electrocardiographic Findings

Electrocardiographic analysis revealed sinus rhythm in 62.5% (n=105) of patients, while atrial fibrillation was documented in 22.6% (n=38). Left ventricular hypertrophy was present in 39.3% (n=66) of cases. ST-segment and T-wave abnormalities were observed in 21.4% (n=36) of patients. Intraventricular conduction disorders were identified in 17.3% (n=29) of cases, with left bundle branch block specifically present in 10.7% (n=18).

Echocardiographic Findings

Echocardiographic findings are summarized in Table 3. Echocardiographic assessment revealed a mean left ventricular ejection fraction (LVEF) of 41.2 ± 13.8%. Based on LVEF classification, heart failure with reduced ejection fraction (HFrEF, LVEF <40%) was the most common phenotype, accounting for 53.6% (n=90) of cases. Heart failure with preserved ejection fraction (HFpEF, LVEF ≥50%) was present in 30.4% (n=51) of patients, while heart failure with mildly reduced ejection fraction (HFmrEF, LVEF 40-49%) represented 16.1% (n=27) of cases.

**Table 3:** Echocardiographic Characteristics (N=168) LVEF: left ventricular ejection fraction; HFrEF: heart failure with reduced ejection fraction; HFmrEF: heart failure with mildly reduced ejection fraction; HFpEF: heart failure with preserved ejection fraction; LV: left ventricular; LVEDD: left ventricular end-diastolic diameter; RV: right ventricular

Parameter	n	%	Mean ± SD
LVEF Classification			
HFrEF (LVEF <40%)	90	53.6	—
HFmrEF (LVEF 40–49%)	27	16.1	—
HFpEF (LVEF ≥50%)	51	30.4	—
Mean LVEF (%)	—	—	41.2 ± 13.8
Structural Abnormalities			
LV dilation (LVEDD >55 mm)	98	58.3	—
RV systolic dysfunction	54	32.1	—
Pulmonary arterial hypertension	74	44.0	—
Valvular Abnormalities			
Mitral regurgitation	80	47.6	—
Tricuspid regurgitation	65	38.7	—

Left ventricular dilation, defined as left ventricular end-diastolic diameter (LVEDD) >55 mm, was observed in 58.3% (n=98) of patients. Right ventricular systolic dysfunction was documented in 32.1% (n=54) of cases. Pulmonary arterial hypertension (estimated systolic pulmonary artery pressure >35 mmHg) was present in 44.0% (n=74) of patients.

Valvular abnormalities were common, with mitral regurgitation detected in 47.6% (n=80) and tricuspid regurgitation in 38.7% (n=65) of patients.

Laboratory Findings

Laboratory investigations revealed anemia (hemoglobin <12 g/dL) in 35.7% (n=60) of patients. Renal insufficiency, defined as serum creatinine >120 μmol/L, was present in 29.2% (n=49) of cases. Hyponatremia (serum sodium <135 mmol/L) was documented in 22.0% (n=37) of patients. Hyperglycemia (fasting blood glucose >7 mmol/L) was observed in 24.4% (n=41) of cases. Elevated C-reactive protein (CRP >6 mg/L), suggesting an inflammatory state, was present in 41.1% (n=69) of patients.

Etiological profile

The etiological distribution of heart failure in our cohort is presented in Table 4. Ischemic cardiomyopathy was the leading cause, accounting for 27.4% (n=46) of cases, followed by hypertensive heart disease in 23.2% (n=39). Dilated cardiomyopathy of idiopathic origin represented 19.6% (n=33) of cases. Valvular heart disease was identified in 14.9% (n=25) of patients. Peripartum cardiomyopathy accounted for 6.0% (n=10) of cases, while rhythm disorder cardiomyopathy (tachycardia-induced) was present in 4.8% (n=8). Other or unknown etiologies comprised 4.2% (n=7) of cases.

**Table 4:** Etiological Distribution of Heart Failure (N=168)

Etiology	n	%
Ischemic cardiomyopathy	46	27.4
Hypertensive heart disease	39	23.2
Dilated cardiomyopathy (idiopathic)	33	19.6
Valvular heart disease	25	14.9
Peripartum cardiomyopathy	10	6.0
Rhythm disorder cardiomyopathy	8	4.8
Other/unknown	7	4.2

Decompensation factors

The precipitating factors for heart failure decompensation identified in our study population are shown in Table 5. Treatment discontinuation (treatment discontinuation) was the most common decompensation factor, present in 35.1% (n=59) of cases. Infections, including pneumonia and sepsis, were identified in 32.7% (n=55) of patients. Uncontrolled hypertension contributed to decompensation in 18.5% (n=31) of cases. Cardiac arrhythmias were responsible for decompensation in 15.5% (n=26) of patients. Dietary non-compliance, particularly excessive salt or fluid intake, was documented in 11.9% (n=20) of cases. Anemia precipitated decompensation in 9.5% (n=16) of patients, while pulmonary embolism was identified in 3.6% (n=6) of cases.

**Table 5:** Decompensation Factors (N=168)

Decompensation Factor	n	%
Treatment discontinuation	59	35.1
Infections (pneumonia, sepsis)	55	32.7
Uncontrolled hypertension	31	18.5
Cardiac arrhythmias	26	15.5
Dietary non-compliance	20	11.9
Anemia	16	9.5
Pulmonary embolism	6	3.6

Therapeutic management

The pharmacological and non-pharmacological management strategies employed in our cohort are summarized in Table 6. Loop diuretics, primarily furosemide, were prescribed to 98.8% (n=166) of patients, reflecting their fundamental role in managing volume overload. Angiotensin-converting enzyme inhibitors (ACEIs) or angiotensin receptor blockers (ARBs) were administered to 76.8% (n=129) of patients. Beta-blockers were prescribed to 64.3% (n=108) of cases. Mineralocorticoid receptor antagonists (MRAs), such as spironolactone, were used in 58.9% (n=99) of patients.

**Table 6:** Therapeutic Management (N=168) ACE: angiotensin-converting enzyme; ARB: angiotensin receptor blocker; SGLT2: sodium-glucose cotransporter-2

Treatment	n	%
Pharmacological Therapy		
Loop diuretics (furosemide)	166	98.8
ACE inhibitors/ARBs	129	76.8
Beta-blockers	108	64.3
Mineralocorticoid receptor antagonists	99	58.9
Anticoagulants	65	38.7
Digoxin	38	22.6
SGLT2 inhibitors	7	4.2
Non-Pharmacological Interventions		
Oxygen therapy	120	71.4

Anticoagulation therapy was administered to 38.7% (n=65) of patients, primarily those with atrial fibrillation or documented thromboembolic risk. Digoxin was prescribed to 22.6% (n=38) of patients, typically for rate control in atrial fibrillation or as inotropic support. Notably, sodium-glucose cotransporter-2 (SGLT2) inhibitors were prescribed to only 4.2% (n=7) of patients, reflecting the limited availability and high cost of these newer agents in Madagascar.

Oxygen therapy was provided to 71.4% (n=120) of patients during the acute phase of hospitalization.

Clinical outcomes

The mean duration of hospitalization was 8.3 ± 4.2 days. Prolonged hospitalization, defined as hospital stay ≥7 days, occurred in 52.4% (n=88) of patients (Table 7).

**Table 7:** Clinical Outcomes (N=168) Percentage calculated among the 152 patients discharged alive and available for follow-up.

Outcome	n	%	Mean ± SD
Mean hospitalization duration (days)	—	—	8.3 ± 4.2
Prolonged hospitalization (≥7 days)	88	52.4	—
Discharge Status			
Favorable outcome (improved)	137	81.5	—
In-hospital mortality	16	9.5	—
Transfer to other facility	6	3.6	—
Discharge against medical advice	9	5.4	—
Follow-up (n=152 discharged alive)			
Rehospitalization	70	41.7*	—

Regarding discharge outcomes, a favorable outcome with clinical improvement was achieved in 81.5% (n=137) of patients. In-hospital mortality occurred in 9.5% (n=16) of cases. Transfer to another healthcare facility was necessary for 3.6% (n=6) of patients, while 5.4% (n=9) of patients left the hospital against medical advice.

Among the 152 patients who were discharged alive and available for follow-up, rehospitalization for heart failure decompensation during the study period occurred in 41.7% (n=70) of cases.

Prognostic factors: multivariate analysis

Factors Associated With Prolonged Hospitalization

Multivariate logistic regression analysis identified right ventricular systolic dysfunction (Table 8) as the only independent factor significantly associated with prolonged hospitalization (≥7 days), with an adjusted odds ratio (ORa) of 6.1 (95% CI: 1.5-23.5; p=0.009). Other factors that showed associations in univariate analysis but did not reach statistical significance in multivariate analysis included atrial fibrillation (ORa=2.1; 95% CI: 0.8-5.4; p=0.128), renal insufficiency (ORa=1.8; 95% CI: 0.7-4.6; p=0.218), and NYHA functional class III-IV (ORa=2.3; 95% CI: 0.9-5.9; p=0.087).

**Table 8:** Multivariate Analysis: Factors Associated With Prolonged Hospitalization (≥7 Days) *Statistically significant (p<0.05); OR: odds ratio; CI: confidence interval; NYHA: New York Heart Association

Factor	Adjusted OR	95% CI	p-value
Right ventricular systolic dysfunction	6.1	1.5–23.5	0.009*
Atrial fibrillation	2.1	0.8–5.4	0.128
Renal insufficiency	1.8	0.7–4.6	0.218
NYHA class III-IV	2.3	0.9–5.9	0.087

Factors Associated With Rehospitalization

Multivariate analysis revealed two independent factors significantly associated with rehospitalization during the study period (Table 9). Atrial fibrillation was associated with a 3.4-fold increased risk of rehospitalization (ORa=3.4; 95% CI: 1.3-29.3; p=0.036). Intraventricular conduction disorders were also independently associated with rehospitalization (ORa=2.3; 95% CI: 1.4-36.6; p=0.013). Heart failure with reduced ejection fraction (HFrEF) and diabetes mellitus showed trends toward increased rehospitalization risk but did not reach statistical significance in multivariate analysis (ORa=1.9; 95% CI: 0.8-4.5; p=0.156 and ORa=1.6; 95% CI: 0.6-4.1; p=0.334, respectively).

**Table 9:** Multivariate Analysis: Factors Associated With Rehospitalization During the Study Period *Statistically significant (p<0.05); OR: odds ratio; CI: confidence interval; HFrEF: heart failure with reduced ejection fraction; LVEF: left ventricular ejection fraction

Factor	Adjusted OR	95% CI	p-value
Atrial fibrillation	3.4	1.3–29.3	0.036*
Intraventricular conduction disorder	2.3	1.4–36.6	0.013*
HFrEF (LVEF <40%)	1.9	0.8–4.5	0.156
Diabetes mellitus	1.6	0.6–4.1	0.334

Factors Associated With In-Hospital Mortality

In multivariate analysis, no factor reached statistical significance for association with in-hospital mortality (Table 10). Renal insufficiency showed a trend toward increased mortality risk (ORa=2.1; 95% CI: 0.5-8.7; p=0.298), as did NYHA functional class IV (ORa=2.8; 95% CI: 0.6-12.4; p=0.184), but neither achieved statistical significance, likely due to the relatively small number of mortality events (n=16) limiting statistical power.

**Table 10:** Multivariate Analysis: Factors Associated With In-Hospital Mortality No factor reached statistical significance (p<0.05). OR: odds ratio; CI: confidence interval; NYHA: New York Heart Association

Factor	Adjusted OR	95% CI	p-value
Renal insufficiency	2.1	0.5–8.7	0.298
NYHA class IV	2.8	0.6–12.4	0.184

## Discussion

This retrospective study of 168 patients hospitalized for heart failure at CHUMT over a three-year period (2021-2023) provides the first comprehensive characterization of heart failure epidemiology, clinical presentation, and outcomes at this major tertiary referral center in eastern Madagascar. Our findings reveal several key observations that have important implications for clinical practice and public health policy in resource-limited settings.

First, heart failure at CHUMT affects a relatively young population (mean age 54 years) with near-equal sex distribution (sex ratio 0.97). Second, hypertension (51.2%), overweight (27.4%), and diabetes mellitus (22.0%) emerged as the predominant cardiovascular risk factors. Third, global (biventricular) heart failure was the most common clinical presentation (75.6%), with ischemic cardiomyopathy (27.4%) and hypertensive heart disease (23.2%) representing the leading etiologies. Fourth, heart failure with reduced ejection fraction (HFrEF) accounted for the majority of cases (53.6%). Fifth, treatment discontinuation (35.1%) and infections (32.7%) were the most frequent decompensation factors. Sixth, while in-hospital mortality was relatively modest (9.5%), the rehospitalization rate during the study period was notably high (41.7%). Finally, multivariate analysis identified right ventricular systolic dysfunction as an independent predictor of prolonged hospitalization, while atrial fibrillation and intraventricular conduction disorders independently predicted rehospitalization.

Age and sex distribution

The mean age of 54 years in our cohort is consistent with the epidemiological pattern observed across sub-Saharan Africa, where heart failure affects substantially younger populations compared to high-income countries. Studies from Gabon (mean age 56 years), Senegal (mean age 58 years), Mali (mean age 52 years), and Togo (mean age 55 years) have reported similar age distributions [6,8,9]. This contrasts markedly with European and North American populations, where the mean age of heart failure patients typically exceeds 70 years [3]. Among the Asian population, the average age is generally in the middle range (mean age 62 years) [10].

The younger age of heart failure onset in sub-Saharan Africa reflects several factors, including higher prevalence of uncontrolled hypertension beginning in early adulthood, limited access to preventive cardiovascular care, delayed diagnosis and treatment of cardiovascular risk factors, higher burden of infectious diseases (including rheumatic heart disease and HIV-associated cardiomyopathy), and potentially genetic predispositions to cardiomyopathies in African populations [5,6]. The socioeconomic implications of heart failure affecting individuals during their most productive years are substantial, resulting in loss of workforce productivity, increased family economic burden, and perpetuation of poverty cycles.

The near-equal sex distribution (50.6% female, 49.4% male) in our study differs from patterns observed in high-income countries, where males typically predominate in younger age groups while females predominate in older age groups due to longer life expectancy [3]. The balanced sex ratio in our cohort may reflect the younger overall age of the population and the high prevalence of hypertensive heart disease, which affects both sexes relatively equally in sub-Saharan Africa [5,8].

Cardiovascular risk factors

Hypertension emerged as the most prevalent cardiovascular risk factor in our cohort (51.2%), consistent with its established role as the leading modifiable risk factor for heart failure in sub-Saharan Africa [5,8]. This finding aligns with data from the Sub-Saharan Africa Survey of Heart Failure (THESUS-HF), which identified hypertension in 45% of heart failure patients across nine African countries [5]. The high prevalence of hypertension in Madagascar reflects inadequate screening programs, limited access to antihypertensive medications, poor medication adherence due to cost and availability issues, insufficient patient education regarding hypertension management, and dietary factors, including high salt intake [11,12].

The substantial prevalence of overweight (27.4%) and obesity (7.1%) in our cohort reflects the ongoing epidemiological transition in Madagascar, characterized by urbanization, dietary changes toward processed foods, and increasingly sedentary lifestyles. This pattern mirrors trends observed across sub-Saharan Africa, where the double burden of undernutrition and overnutrition coexists [13]. The prevalence of diabetes mellitus (22.0%) in our heart failure cohort is notably higher than general population estimates for Madagascar, underscoring the strong bidirectional relationship between diabetes and heart failure [1,13].

Smoking prevalence (14.9%) in our cohort was lower than rates reported in some African countries, potentially reflecting cultural factors and the relatively high cost of tobacco products in Madagascar. However, the true prevalence may be underestimated due to underreporting, particularly among women, for whom smoking carries a greater social stigma.

Clinical presentation

Dyspnea was the predominant presenting symptom (94.6%), consistent with its recognition as the cardinal symptom of heart failure across all populations [1,4]. The high proportion of patients presenting with advanced symptoms (NYHA class III-IV in 67.9%) reflects delayed presentation to healthcare facilities, a common pattern in resource-limited settings, attributable to limited health literacy, geographic barriers to healthcare access, financial constraints, and initial presentation to traditional healers [5,6].

The predominance of global (biventricular) heart failure (75.6%) in our cohort is consistent with findings from other African studies and reflects the advanced stage of disease at presentation [6,8,9]. This contrasts with high-income countries, where earlier diagnosis often allows identification of predominantly left-sided or right-sided heart failure before progression to global involvement [7]. The high prevalence of global heart failure has important therapeutic implications, as these patients typically require more intensive diuretic therapy and have a higher risk of complications, including renal dysfunction and electrolyte disturbances [1,14].

Echocardiographic profile

The predominance of HFrEF (53.6%) in our cohort is consistent with data from other sub-Saharan African studies, where HFrEF typically accounts for 50-70% of heart failure cases [5,6,8]. This contrasts with contemporary data from high-income countries, where HFpEF now represents approximately 50% of heart failure cases, reflecting aging populations and increasing prevalence of metabolic comorbidities [3,7]. The mean LVEF of 41.2% in our cohort indicates substantial systolic dysfunction and highlights the advanced nature of the disease at presentation.

The high prevalence of left ventricular dilation (58.3%) and right ventricular systolic dysfunction (32.1%) further underscores the severity of cardiac remodeling in our population. Right ventricular dysfunction, which emerged as an independent predictor of prolonged hospitalization in our multivariate analysis, is increasingly recognized as a critical prognostic marker in heart failure [15]. The presence of right ventricular dysfunction indicates advanced disease, portends worse outcomes, and necessitates more intensive monitoring and management [15].

Pulmonary arterial hypertension (44.0%) and significant valvular regurgitation (mitral regurgitation 47.6%, tricuspid regurgitation 38.7%) were common findings, reflecting the hemodynamic consequences of chronic left ventricular dysfunction [5,6].

Etiological spectrum

The etiological profile of heart failure in our cohort reveals important differences from both high-income countries and some other African settings. Ischemic cardiomyopathy emerged as the leading etiology (27.4%), followed by hypertensive heart disease (23.2%) and dilated cardiomyopathy (19.6%). This pattern differs from many sub-Saharan African studies, where hypertensive heart disease typically predominates [5,8]. For example, the THESUS-HF registry identified hypertensive heart disease as the leading cause in 45% of cases, while ischemic heart disease accounted for only 8% [5].

The relatively high prevalence of ischemic cardiomyopathy in our cohort may reflect several factors specific to the Toamasina region and CHUMT’s referral patterns, including increasing prevalence of atherosclerotic risk factors (diabetes, dyslipidemia, smoking) in urban Madagascar, improved diagnostic capabilities at CHUMT allowing better identification of ischemic etiology, referral bias with more complex ischemic cases being referred to the tertiary center, and potentially underdiagnosis of ischemic heart disease in other African studies with limited access to coronary angiography [5,6].

The substantial proportion of dilated cardiomyopathy (19.6%) likely includes cases of idiopathic dilated cardiomyopathy as well as undiagnosed secondary causes such as viral myocarditis, HIV-associated cardiomyopathy, and nutritional deficiencies. The prevalence of peripartum cardiomyopathy (6.0%) is consistent with rates reported in other African studies and reflects the high maternal cardiovascular risk in sub-Saharan Africa [5,6].

Decompensation factors

Treatment discontinuation emerged as the most common precipitating factor for heart failure decompensation (35.1%), highlighting a critical challenge in chronic disease management in resource-limited settings. This finding is consistent with previous Malagasy data from Antananarivo, where treatment discontinuation was identified as a major decompensation factor [13]. Multiple barriers contribute to treatment discontinuation in Madagascar, including medication cost and availability issues, inadequate patient education regarding the chronic nature of heart failure, complex medication regimens with multiple daily doses, lack of social support systems, geographic barriers to pharmacy access, and absence of structured follow-up programs [12,13].

A systematic review of medication adherence in heart failure patients in sub-Saharan Africa identified adherence rates ranging from 23% to 87%, with cost being the most frequently cited barrier [9]. Addressing treatment discontinuation requires multifaceted interventions, including subsidized medication programs, simplified once-daily medication regimens where possible, structured therapeutic education programs, community health worker support for medication adherence, and establishment of patient support groups [9].

Infections (32.7%) emerged as the second most common decompensation factor, reflecting the high burden of infectious diseases in Madagascar and the increased susceptibility of heart failure patients to infections due to pulmonary congestion, immunosuppression from chronic illness, and malnutrition. Pneumonia was the most common infection, followed by urinary tract infections and sepsis. Prevention strategies should include pneumococcal and influenza vaccination (where available), early recognition and treatment of infections, and patient education regarding infection prevention [1,5].

Therapeutic management

The high utilization rates of guideline-directed medical therapy in our cohort, including loop diuretics (98.8%), ACEIs/ARBs (76.8%), beta-blockers (64.3%), and mineralocorticoid receptor antagonists (58.9%), demonstrate good adherence to contemporary heart failure management guidelines despite resource constraints [1,4]. These rates compare favorably with data from other African centers and reflect the commitment of CHUMT cardiology staff to evidence-based practice [5,6].

However, the very low utilization of SGLT2 inhibitors (4.2%) represents a significant gap in optimal heart failure management. SGLT2 inhibitors have emerged as a cornerstone of HFrEF therapy, demonstrating substantial reductions in cardiovascular mortality and heart failure hospitalizations in landmark trials [1]. The limited use of SGLT2 inhibitors at CHUMT reflects their high cost, limited availability in Madagascar, lack of inclusion in national essential medicines lists, and insufficient awareness among some clinicians regarding their benefits in heart failure [1]. Advocacy for improved access to SGLT2 inhibitors through generic formulations, international assistance programs, and national policy changes should be a priority for cardiovascular health in Madagascar.

The high utilization of oxygen therapy (71.4%) reflects the severity of presentation in our cohort, with most patients experiencing significant dyspnea and hypoxemia at admission. Non-pharmacological interventions, including dietary sodium restriction, fluid restriction, and patient education, were implemented, though systematic documentation of these interventions was limited in the retrospective medical records.

Clinical outcomes and prognostic factors

The in-hospital mortality rate of 9.5% in our cohort falls within the range reported in other sub-Saharan African studies (9-20%) and is substantially higher than rates observed in high-income countries (4-7%) [5,7]. This mortality differential reflects multiple factors, including more advanced disease at presentation, higher burden of comorbidities, limited access to advanced therapies (cardiac resynchronization therapy, implantable cardioverter-defibrillators, mechanical circulatory support), and resource constraints affecting intensive care capabilities [5,6].

The rehospitalization rate of 41.7% is notably high and represents a major challenge for both patients and the healthcare system. This rate is consistent with or higher than rates reported in other African studies and substantially exceeds contemporary rates in high-income countries (20-30%) [5,7]. The high rehospitalization rate reflects inadequate post-discharge support systems, treatment discontinuation due to cost and availability, insufficient patient education regarding self-care and early warning signs, limited access to outpatient cardiology follow-up, and persistence of decompensation factors (particularly infections and uncontrolled hypertension) [11,12].

Our multivariate analysis identified three independent prognostic factors with important clinical implications. Right ventricular systolic dysfunction emerged as a powerful predictor of prolonged hospitalization (ORa=6.1), consistent with its established role as a marker of advanced heart failure and poor prognosis [15]. This finding underscores the importance of systematic echocardiographic assessment of right ventricular function in all heart failure patients and should prompt more intensive monitoring and management strategies for patients with right ventricular dysfunction [15].

Atrial fibrillation was independently associated with rehospitalization (ORa=3.4), reflecting its association with worse hemodynamics, increased thromboembolic risk, and challenges in rate and rhythm control [16][17]. Patients with atrial fibrillation require careful attention to anticoagulation (when not contraindicated), optimal rate control, and consideration of rhythm control strategies [17]. Intraventricular conduction disorders were also independently associated with rehospitalization (ORa=2.3), potentially reflecting more advanced structural heart disease and suggesting that selected patients might benefit from cardiac resynchronization therapy if resources permit [1,4].

The absence of statistically significant independent predictors of in-hospital mortality in our multivariate analysis likely reflects the relatively small number of mortality events (n=16), limiting statistical power. Larger prospective studies are needed to better characterize mortality risk factors in the Malagasy heart failure population.

Strengths and limitations

Strengths

This study has several notable strengths. First, it represents the first comprehensive characterization of heart failure epidemiology, clinical presentation, and outcomes at CHUMT, providing essential baseline data for quality improvement initiatives and health policy planning. Second, all cases were confirmed by echocardiography, ensuring diagnostic accuracy and allowing detailed characterization of cardiac structure and function. Third, the three-year study period provides a substantial sample size and reduces the impact of seasonal or temporal variations. Fourth, the use of multivariate analysis allowed identification of independent prognostic factors while controlling for confounding variables. Fifth, the study adhered to contemporary international guidelines for heart failure definition and classification [1].

Limitations

Several limitations should be acknowledged. First, the retrospective design relies on medical record documentation, which may be incomplete or inconsistent, potentially leading to underestimation of some variables. Second, as a single-center study at a tertiary referral hospital, our findings may not be fully generalizable to primary or secondary care settings in Madagascar, and referral bias may have resulted in overrepresentation of more severe or complex cases. Third, data on some important prognostic markers, particularly natriuretic peptides (B-type natriuretic peptide (BNP)/N-terminal pro-B-type natriuretic peptide (NT-proBNP)), were available for only a small subset of patients due to limited laboratory capacity and high cost, precluding their inclusion in prognostic analyses. Fourth, information on medication adherence, quality of life, and functional capacity at follow-up was not systematically collected. Fifth, the relatively small number of mortality events (n=16) limited statistical power for identifying independent predictors of in-hospital mortality. Finally, data on some potentially important variables, such as detailed dietary patterns, physical activity levels, and socioeconomic indicators, were not systematically available in medical records.

## Conclusions

This study provides the first comprehensive characterization of heart failure epidemiology, clinical presentation, and outcomes at the University Hospital Center Morafeno Toamasina (CHUMT), a major tertiary referral center in eastern Madagascar. Heart failure at CHUMT predominantly affects a relatively young population with near-equal sex distribution, most commonly presenting as global (biventricular) heart failure. Hypertension, overweight, and diabetes mellitus emerged as the leading modifiable cardiovascular risk factors, while ischemic cardiomyopathy and hypertensive heart disease were the principal etiologies. Treatment discontinuation and infections were identified as the most frequent precipitating factors for decompensation, highlighting critical targets for intervention through structured therapeutic education and infection prevention strategies. Multivariate analysis identified right ventricular systolic dysfunction as an independent predictor of prolonged hospitalization, and atrial fibrillation and intraventricular conduction disorders as independent predictors of rehospitalization. Although in-hospital mortality was comparable to rates reported across sub-Saharan Africa, the rehospitalization rate was notably high, underscoring the urgent need for robust post-discharge support programs. Addressing the growing burden of heart failure in Madagascar requires a comprehensive approach encompassing primary prevention, early diagnosis, optimized therapeutic management, and structured patient education, supported by evidence-based cardiovascular health policy.
